# Systemic chemotherapy plus cetuximab after complete surgery in the treatment of isolated colorectal peritoneal carcinoma: COCHISE phase II clinical trial

**DOI:** 10.1186/s13104-019-4476-9

**Published:** 2019-07-22

**Authors:** Serge Evrard, Grégoire Desolneux, Carine Bellera, Thomas Esnaud, Yves Bécouarn, Denis Collet, Najim Chafai, Francois Marchal, Laurent Cany, Emilie Lermite, Michel Rivoire, Simone Mathoulin-Pélissier

**Affiliations:** 1 Digestive Tumours Unit, Institut Bergonié, Comprehensive Cancer Centre, 229 Cours de l’Argonne, 33076 Bordeaux, France; 2 Clinical Research and Clinical Epidemiology Unit (ISO 9001 Certified), Institut Bergonié, Comprehensive Cancer Centre, 229 Cours de l’Argonne, 33076 Bordeaux, France; 3 INSERM CIC-EC 14.01 (Clinical Epidemiology), Bordeaux, France; 4 Department of Visceral Surgery, CHU Bordeaux, Haut Levêque, 33000 Pessac, France; 5 Department of Visceral Surgery, Hôpital Saint Antoine, 184 Rue du Faubourg Saint-Antoine, 75012 Paris, France; 6 Department of Visceral Surgery, CHU Nancy, Rue du Morvan, 54500 Vandœuvre-lès-Nancy, France; 7 Clinique Francheville, 34 Boulevard de Vesone, 24000 Périgueux, France; 8 Department of Visceral Surgery, CHU Angers, 4 Rue Larrey, 49100 Angers, France; 9 Department of Surgical Oncology, Centre Léon Bérard, Comprehensive Cancer Centre, 28 Promenade Léa et Napoléon Bullukian, 69008 Lyon, France; 10 Université de Lyon, 92 Rue Pasteur, 69007 Lyon, France; 11 Univ. Bordeaux, 146 rue Léo Saignat, 33000 Bordeaux, France

**Keywords:** Colorectal cancer, Peritoneal carcinomatosis, Cytoreductive surgery, *KRAS* gene, Hyperthermic intraperitoneal chemotherapy

## Abstract

**Objective:**

The primary objective of this non-randomised phase II study was to evaluate the combination of systemic chemotherapy plus cetuximab after complete cytoreductive surgery (CCS) for treatment of isolated colorectal peritoneal carcinoma (CRPC). This multicentre, prospective phase II clinical trial was conducted in seven national cancer referral centres, however research published during study recruitment indicated cetuximab treatment as ineffective in patients with mutated KRAS genes, leading to an additional exclusion criterion to the current protocol, excluding patients with mutated KRAS genes. This significantly impacted recruitment and the study did not achieve the necessary recruitment of 46 patients.

**Results:**

Fourteen patients underwent CCS and were included in the study, however one did not provide informed consent and another received only one cycle of chemotherapy leading to 12 patients in the per protocol population for analysis. Adjuvant Folfox Cetuximab was administered when CCS was achieved for patients > 18 years with histologically proven CRPC and no other metastatic disease (liver, lungs, lymphadenopathy, etc.). CRPC median index was 5.00 (range: 1–17). Median PFS was 12.3 months [95% CI (3.7–28.2)] with 8.3% [95% CI (0.5–31.1)] and 0% PFS at 3 and 5 years respectively. Median OS was 43.4 months [95% CI (16.8–60)].

*Trial registration* Clinical Trials NCT00766142, October 3, 2008. Retrospectively registered

## Introduction

Peritoneal carcinomatosis (PC) occurs in around 13% of colorectal cancer cases [1]. PC can be a sole metastasis or associated with other organs such as the liver. While long considered only in non-curative settings, complete cytoreductive surgery (CCS) with hyperthermic intraperitoneal chemotherapy (HIPEC) has been proposed as an additional treatment to systemic chemotherapy [2]. Surgeons specialising in PC have progressively settled for HIPEC as the standard treatment, although the true clinical impact of the addition of HIPEC is still under scrutiny [3, 4]. The only prospective evaluation of HIPEC so far was a Dutch study in 2003, comparing CCS plus HIPEC versus palliative chemotherapy [5]. While CCS + HIPEC gave better results than palliative chemotherapy, the authors caution that the actual impact of HIPEC cannot be disentangled from the effectiveness of CCS.

The lack of prospective clinical data on colorectal PC (CRPC) prompted us to design a non-randomised phase II study to evaluate a strategy based only on complete surgery (R0/R1) and systemic treatment without HIPEC—the COCHISE study (NCT00766142). At the time of protocol writing in 2007, the optimal regimen to treat colorectal cancer was FOLFOX-4 plus cetuximab [6], with response rates of 81%, including 10% of complete responses and a median progression-free survival (PFS) of 12.3 months. At that time, the efficacy of cetuximab in the adjuvant setting was under investigation and the predictive/prognostic value of *KRAS* status and its effect on treatment outcomes was unknown. We report here, the results of the COCHISE study. Despite the fact that this trial failed to accrue the required number of patients, it provides prospective data on progression-free survival (PFS) and overall survival (OS) rates for CRPC treated by CCS without HIPEC.

## Main text

### Patients and methods

The primary objective of this multicentre, phase II clinical trial was to evaluate the efficacy, in terms of PFS rate at 3 years, of the combination of systemic chemotherapy plus cetuximab after complete surgery in the treatment of isolated CRPC, without HIPEC. PFS was defined as the delay between the date of inclusion and the date of progression or death (of any cause), whichever occurred first.

The secondary objectives were to assess PFS rate at 5 years and overall survival (OS) rate at 3 and 5 years, of the aforementioned combinatorial treatment. OS was defined as the delay between the date of inclusion and the date of death (of any cause). Furthermore, we assessed the overall tolerance (mortality, morbidity) of systemic chemotherapy with cetuximab in comparison with a therapeutic strategy combining maximal surgery, systemic chemotherapy and cetuximab.

#### Inclusion and exclusion criteria

Patients > 18 years with histologically proven CRPC and no other metastatic disease (liver, lungs, lymphadenopathy, etc.) who signed a written informed consent and had French social security were included in the study. There was no upper age limit, but an oncogeriatric assessment was required for patients > 75 years of age. A major amendment was made to the protocol following data presented at the 2008 ASCO congress indicating that cetuximab treatment is ineffective in patients with a mutated *KRAS* gene. This information led to the temporary suspension of the study and an additional inclusion criterion was introduced to recruit only patients with wildtype *KRAS*. The gene sequence was centrally verified at the Department of Pathology, Institut Bergonié. Furthermore, the inclusion period was extended and additional consent was required for 5 patients already included for retrospective *KRAS* status determination.

It should also be noted that, at the expressed request of Merck KGaA, the study was initially designed in 2007 to position cetuximab as an adjuvant treatment. This excluded the principle of first-line chemotherapy, which was nevertheless preferred by medical oncologists for carcinomatosis management. Consequently, it has not been possible to convince enough participating oncologists to take up surgical management first, followed by adjuvant chemotherapy. Furthermore, many patients referred to our centre for surgical treatment of their PC had already received chemotherapy. Finally, cetuximab was considered not efficient in adjuvant setting [7] and hence, it was possible to negotiate a second amendment introducing a perioperative chemotherapy protocol. Unfortunately, the dynamic of the trial was broken and it was not possible to include new patients. (Only 16 patients (KRAS mutated and non-mutated combined) had been included since 15/05/2007 in 7 participating national investigative centres and no patients were included after 18/10/2011. Due to these recruitment problems the study was closed prematurely on 17/09/2013. One of the main reasons is as many as 48% [8] of patients with isolated PC of colorectal origin carry a mutated *KRAS* gene).

### Surgery

Prior to surgery, all patients with colorectal cancer who met the inclusion criteria for isolated PC were pre-selected. All patients signed a preliminary consent authorizing the investigator to collect and analyse information on disease and surgery. The surgical procedure started with an exploratory laparotomy followed, if possible, by an R0 complete resection of PC. All participating centres followed a homogeneous and standardized procedure and were selected for their experiences after they achieved their learning curve [9]. Patients were installed in a prone position to allow access to the rectum. A xipho-pubic laparotomy was carried out and a circular retractor installed. The Sugarbaker peritoneal carcinomatosis index (PCI) [10] was used to assess the extent of PC with a rough limit positioned at 20/39. We proceeded with a complete resection of all macroscopic disease considered to be possible within a maximum of 10 h of surgery limiting morbidity and mortality. The operative field was washed hourly with a hot saline mix in order to maintain the body temperature and to retrieve cellular debris. Adverse events were recorded and graded according to the common terminology criteria for adverse events (CTCAE) v3.0 from NCI (Table 2). All patients in a good postoperative condition i.e. without any serious complications received FOLFOX plus cetuximab, starting within 4 to 8 weeks.

#### Chemotherapy

Patients completely resected (R0) for their PC were proposed to enter the study by signing the informed consent.

### Statistical analyses

The trial was initially designed assuming 50% 3-year PFS rate, with anticipated 95% confidence interval (95% CI) ranging from 36 to 64% requiring 46 patients. The ITT population was initially defined as all patients who underwent surgery or received at least one cycle of chemotherapy. We subsequently defined the PP population as all eligible patients, with wild type KRAS, who underwent surgery and received at least 6 cycles of chemotherapy.

PFS and OS were analysed using the Kaplan–Meier method. Survival data were censored at the date of last contact in case of lost-to-follow-up. For PFS analysis, patients who were alive and progression-free were censored at the date of last follow-up. Median survival rates with their 95% CI as well as 3- and 5-year survival rates were reported. Post-operative mortality (within 30 days), surgical morbidity and serious adverse events (expected and unexpected) were described using frequencies and rates according to the NCI CTCAE (v3.0) scale.

All analyses were conducted using SAS 9.2 software (SAS Institute, Cary, NC). ClinicalTrials.gov NCT00766142.

### Results

#### Patient characteristics

Eighteen patients were pre-selected between September 2007 and July 2011, including 14 patients with wild type *KRAS*. Of these, 14 were resected. One patient did not provide subsequent informed consent and one patient received only one cycle of chemotherapy leading to 13 and 12 patients in the ITT and PP population respectively. As results did not significantly differ between the two populations, only data on the PP population are reported (Table 1). Median PCI was 5.00 (range: 1–17).

**Table 1:** Patient characteristics

	PP population	
	N	%
Women	2	16.7
Men	10	83.3
Median age (years)	63.00	
Median height (cm)	171.00	
Median weight (kg)	76.00	
General status (WHO)		
Not available	6	50.0
WHO 0 (CV 100%)	1	8.3
WHO 1 (CV 90–80%)	5	41.7
Clinical examination		
Not available	2	16.7
Abnormal	0	0.0
Normal	9	75.0
Not performed	1	8.3

*PP population* per protocol population

#### Primary endpoint

**Fig. 1:**
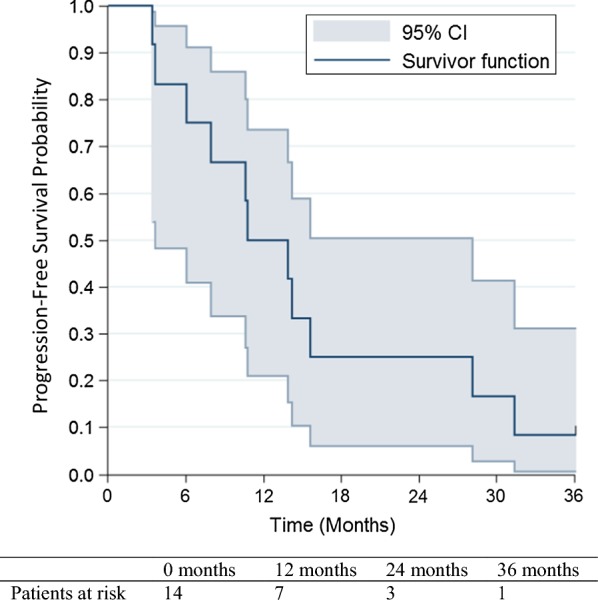
Per protocol population 3-year progression-free survival

Median PFS was 12.3 months [95% CI (3.7–28.2)] and the 3- and 5-year rates were 8.3% [95% CI (0.5–31.1)] and 0%, respectively (Fig. 1).

#### Secondary endpoints

Median OS was 43.4 months [95% CI (16.8–60)] and the 3- and 5-year OS rates were 58.3% [95% CI (27.0–80.1)] and 38.8% [95% CI (12.6–65.0)], respectively.

Regarding the 30-day post-operative morbidity, 8 adverse events (AEs) (4 patients) were reported in the PP population, including three grade 3 and one grade 4 AEs (Table 2). During and after chemotherapy, 115 AEs were reported including 94 grade 1–2 AEs, 15 grade 3–4 AEs and one grade 5 AE. Of those, 46 AEs were treatment-related, 44 had a probable relationship and 25 were unrelated to the treatment. Finally, 10 serious AEs (SAE) were observed (6 patients) and no unexpected severe AEs were reported.

**Table 2:** 30-day post-operative morbidity: adverse events reported during and within 30 days of surgery according to NCI-CTC AE v 3.0 (per protocol population)

Description	AE	Grade	Surgery-related	SAE
Haemorrhage/bleeding	Pelvic haematoma	Grade 2	Probable	No
Haemorrhage/bleeding	Haemorrhage	Grade 3	Certain	Yes, expected
Constitutional symptoms^a^	Weight loss	Grade 3	Certain	No
Renal/genitourinary^a^	Left urethral fistula	Grade 3	Certain	Yes, expected
Neurology^a^	Depression	Grade 4	N/A	No
Surgery/intra-operative injury^a^	Intra-peritoneal collection	Grade 1	Certain	No
Gastrointestinal^b^	Anastomotic colon fistula	Grade 2	Certain	No
Cardiac arrhythmia^b^	Tachycardia	Grade 2	N/A	No

*AE* adverse events, *SAE* severe adverse events

^a^These 4 AE were for the same patient

^b^These two AE were for the same patient

### Discussion

The objective of the COCHISE trial was to evaluate the management of CRPC on the hypothesis that therapeutic efficacy could be achieved with complete radical surgery associated with the best systemic treatment. In other words, we hypothesized that HIPEC is a futile addition in CRPC treatment. The failure to accrue sufficient patient numbers also observed in other attempts of prospective series of colorectal peritoneal carcinomatoses [11] can be explained by three reasons. First, it was reported in 2008 [12], just after the start of our trial, that cetuximab is only efficient in patients with a wild type *KRAS* gene. However, many patients with isolated CRPC carry mutations in the *KRAS* gene and hence, were excluded. Second, as noted earlier, many oncologists were reluctant to refer patients for surgery who had not responded to initial chemotherapy, which was a major exclusion criterion for the study. Third, by excluding patients with extra-peritoneal metastases (liver, lung, etc.), we hyper-selected a rare population which further increased the accrual difficulties. Consequently, this population had lower PCI (median = 5) with a slightly better prognosis compared to CRPC series with additional extra-peritoneal metastases.

Despite these setbacks, the COCHISE study provides useful prospective data. Our hypotheses were built on a recently published retrospective series with OS rates of 45.5% and 29.6% at 3 and 5 years, a series of 50 patients who were given different perioperative chemotherapy protocols [13]. In that series, 26 patients out of 50 also had extra hepatic metastases and the median PCI was 8 which may explain the slight difference. Even though underpowered and with broad CIs, the OS rates in our RP of 53.8% at 3 years and 35.9% at 5 years are consistent with the hypothesis set out in the original design of the study. The OS is comparable to the retrospective data published with HIPEC varying from 14 [14] to 60 months [15]. Interestingly, our observed median OS of 43.4 months without HIPEC is similar to the recently presented data of Prodige 7 trial (ASCO 2018), 41.7 months OS with HIPEC versus 41.2 without. Of note, the toxicity pattern due to the FOLFOX regimen was similar to what was reported in the literature.

The notion that HIPEC is futile to treat CRPC is not new, originating from the tenuity of the initial claims, as well as the lack of rigor in expert opinions [16]. HIPEC is an old treatment (established in the early eighties), conceived at the time when only very few systemic cytotoxic drugs with low efficacy such as mitomycin C were available. The idea was to increase the concentration of the drug by local exposure and to increase the local temperature to boost its pharmacodynamic properties. The same concept was used to provide a ground for isolated organ perfusion, like limb perfusion with TNF-alpha and melphalan [17]. Furthermore, in oncological settings, a new treatment generally has to prove its efficacy first in the palliative setting before being tested as an adjuvant. HIPEC followed exactly the opposite path: from being inactive on major macroscopic deposits, it was used further on small residues with limited additional success. Finally, the indications were further reduced to only after R0 surgery, which is the best way to hide the purported effectiveness of HIPEC behind the effectiveness of surgery. Another paradox is that intraperitoneal chemotherapy is expected to provide better absorption through the peritoneal sheets, when CCS requires retrieving all tumoral deposits (R0), sometimes even with the excision of wide peritoneal surfaces. Consequently, following surgery, it is the aponeurosis or the nude muscles that are exposed to chemotherapy and not the peritoneum. Ultimately, HIPEC violates the intangible evidence that no cytotoxic applied only once is able to sterilise a tumoral deposit, especially when the cytotoxic like oxaliplatin or mitomycin C have very low in vitro efficacy against the given cancer.

## Limitations

Soon after the trial commenced, it was reported that cetuximab is only efficient in patients with wild type *KRAS* gene. This led us to amend the accrual according to the *KRAS* gene status. Unfortunately, many patients with CRPC carry mutations in this gene. Hence, the required number of patients originally set out could not be attained. As such, the results of the study are considered underpowered and should be considered as promising prospective evidence for PFS and OS rates that require further investigation.

The survivals of patients prospectively treated and followed-up without HIPEC are consistent with those retrospectively collected with HIPEC [13]. And above all, our observed median OS of 43.4 months without HIPEC is similar to the recently presented data of Prodige 7 trial (ASCO 2018), which does not show any difference in OS with or without HIPEC. We thus put forward a further nail in the coffin on the debate on HIPEC’s role in CRPC. As an experimental treatment, HIPEC should not be proposed to patients outside of clinical trials.

## Data Availability

The datasets generated and/or analysed during the current study are not publicly available due to restrictions by the French National Commission for Information and Liberties (CNIL) but are available from the corresponding author on reasonable request.

## Abbreviations

CCS: complete cytoreductive surgery
CRPC: isolated colorectal peritoneal carcinoma
PFS: progression-free survival
OS: overall survival
HIPEC: hyperthermic intraperitoneal chemotherapy
PP: per protocol
ASCO: American Society of Clinical Oncology
NCI CTCAE: National Cancer Institute Common Terminology Criteria for Adverse Events
AE: adverse events
PCI: Peritoneal Cancer Index
